# Cost-Effectiveness Analysis of Topical Treatments for Actinic Keratosis in Italian National Health System

**DOI:** 10.3390/jcm13216312

**Published:** 2024-10-22

**Authors:** Mariafrancesca Hyeraci, Gianluca Pistore, Francesco Ricci, Francesco Moro, Giovanni Di Lella, Elena Dellambra, Damiano Abeni, Luca Fania

**Affiliations:** IDI-IRCCS, Dermatological Research Hospital, Via dei Monti di Creta 104, 00167 Rome, Italy; gianluca@gianlucapistore.com (G.P.); f.ricci@idi.it (F.R.); f.moro@idi.it (F.M.); g.dilella@idi.it (G.D.L.); e.dellambra@idi.it (E.D.); d.abeni@idi.it (D.A.); l.fania@idi.it (L.F.)

**Keywords:** actinic keratosis, cost-effectiveness, Italian healthcare system, 5-fluorouracil, diclofenac, imiquimod, tirbanibulin

## Abstract

**Introduction:** Actinic keratosis (AK) is a widespread pre-cancerous skin condition that may evolve to squamous cell carcinoma, a non-melanoma skin cancer, which is able to become locally invasive and metastatic. Thus, it is important to treat AK. **Methods**: We conducted a cost-effectiveness analysis for the field-directed therapeutic approaches: local application of drugs containing 5-fluorouracil, both alone at a 4% concentration and associated to 10% salicylic acid at a 0.5% concentration (0.5% 5-FU + 10% SA cut. sol.); diclofenac-hyaluronic acid gel; imiquimod, both at 3.75% and 5% (5% IMQ cream) concentrations; and tirbanibulin ointment. The effectiveness data were abstracted from the literature. The cost-effectiveness analysis was performed by considering the prices reported by Agenzia Italiana del Farmaco (AIFA) for each medicine. **Results**: We obtained the total cost for each treatment by computing the cost of a single treatment for its duration. Application of 0.5% 5-FU + 10% SA cut. sol. appeared as the most convenient approach, as it was more effective and less expensive than all treatments except for 5% IMQ cream. For this last option, the incremental cost/effectiveness ratio analysis showed that a modest gain in effectiveness has a cost of EUR 7.94, therefore making it less cost effective.

## 1. Introduction

Actinic keratosis (AK) is a very common pre-cancerous lesion arising from the abnormal proliferation of keratinocytes in skin areas chronically damaged by exposure to ultraviolet (UV) rays, affecting about 40% of the adult population [1,2]. In the light spectrum, wavelengths corresponding to UV-B (290–320 nm) are considered as the most damaging for skin health, and the ozone layer is reported as the main natural filter of UV-B light. Unfortunately, the thickness of ozone layer is gradually reducing, and this event represents the main cause of the continuously increasing incidence of AK disease [3]. The main risk factors for AK include exposure to UV rays, age, male sex, Fitzpatrick skin type I–II, immunosuppression, and previous diagnosis of AK or non-melanoma skin cancer (NMSC). On the contrary, the use of both topic and systemic photoprotection and a body mass index ≥ 25.0 kg/m^2^ are considered as protective factors [4,5].

The main complication associated with AK is the progression to squamous cell carcinoma (SCC) [6], a cutaneous carcinoma that can become locally invasive and metastatic [7]. Since it is impossible to exactly predict whether an AK lesion will transform into SCC, it is very important to treat both AKs and the surrounding area, which may host subclinical manifestations of a “field cancerization” [8].

The different therapeutic approaches are finalized to treat both clinical and subclinical lesions, including the entire cancerous field. The currently available therapeutic options should be divided into two groups, i.e., the lesion- and the field-targeted treatments. Within the first group are cryotherapy, laser therapy, curettage, and surgery. Among the field-targeted treatments are the topical application of drugs containing 5-fluorouracil (5-FU), both alone at a 4% concentration and associated with 10% salicylic acid (SA) at a 0.5% concentration; diclofenac-hyaluronic acid (DIC-HA); imiquimod (IMQ), both at a 3.75% and 5% concentrations; tirbanibulin (Tirb); and photodynamic therapy (PDT) [6]. The choice of treatment depends on the characteristics of both the lesions and patients. For instance, the presence of comorbidities (i.e., cardiovascular or neurological diseases, diabetes, etc.) must be considered to select the most appropriate option and to increase the patient’s adherence to treatment [9]. Moreover, the impact on daily activities, especially for lesions located in visible parts of the body, should be considered during the choice, along with the patient’s ability to correctly self-apply the topical agents at home.

In Italy, a very high incidence has been reported for AK, involving around 27% of the Italian population over 30 [10]. As AK is an in situ skin cancer whose main complication is the development of infiltrated SCC, it is urgent to treat every patient affected by AK. We can easily understand that the huge number of patients renders AK disease a heavy burden for the Italian NHS. Therefore, the aim of this study was to perform a cost-effectiveness analysis of the currently available therapeutic options among the topical treatments for the management of AK within the framework of the Italian NHS.

## 2. Materials and Methods

The pharmacoeconomic analysis was conducted according to the CEA method, which allows to compare the cost/effectiveness (C/E) ratios of therapies that have different costs and success rates in achieving the same desired outcome. The C/E ratio is given as the ratio between the average cost per patient of the treatment in the time period considered and the average efficacy per patient of the treatment in the same time period, i.e., the average cost of the unit of outcome per patient. For the ideal treatment, which should be very effective and inexpensive, the C/E ratio is very low, proximal to 0.

### 2.1. Treatments Considered

The cutaneous solution 0.5% 5-FU + 10% SA (0.5% 5-FU + 10% SA cut. sol.) is approved and available on the market in Europe for the treatment of grade I/II AK in adult immunocompetent patients. It consists of the combination of two different drugs: 5-FU at a 0.5% concentration and SA at a 10% concentration. The latter is a keratolytic agent and works as an adjuvant towards 5-FU by increasing its penetration into the skin. 5-FU is the proper chemotherapeutic agent [11]. As an uracil analogue, with a fluorine atom at the C-5 position in place of a hydrogen, it enters into the cells through the facilitated transport mechanism used by uracil [12]. It works as a prodrug, and once in the cells, it is enzymatically converted into its active metabolites, which provokes the irreversible inhibition of thymidylate synthase target enzyme. The latter regulates the sole pathway that provides the de novo source of deoxythymidine monophosphate (dTMP). Depletion of dTMP results in deoxynucleotide pool imbalances (in particular, the dATP/dTTP ratio) that disrupt DNA synthesis, finally resulting in lethal DNA damage [13].

Additionally, 4% 5-FU cream is approved for the treatment of grade I and II AK on the face, ears, or scalp in adults. It contains the above-mentioned and -described 5-FU at a 4% concentration.

DIC 3% + HA 2.5% gel is approved for the treatment of AKs in adult patients. It contains DIC and HA at a 3% and 2.5% concentration, respectively. DIC, belonging to the non-steroidal anti-inflammatory drugs family (NSAIDs), is able to inhibit both 1 and 2 isoforms of cyclooxygenase (COX), an enzyme that catalyzes the conversion of arachidonic acid into prostaglandins E2 [14]. Although these molecules are mainly involved in inflammatory process, they can stimulate cell proliferation and motility, induce angiogenesis, and inhibit immune surveillance and apoptosis [15]. Interestingly, enhanced levels of COX2 have been found in both AK [16] and SCC [17]. Furthermore, 3.75% IMQ cream and 5% IMQ cream are two creams approved for the treatment of AK on the face and balding parts of the scalp in immunocompetent adults when other treatments cannot be used or are less appropriate [18]. The active drug of both the creams is IMQ at a 3.75% and 5% concentration, respectively. IMQ is an immunomodulator agent able to stimulate both the innate and the acquired immune response. In detail, IMQ is an agonist of Toll-like receptors 7 and 8, and the interaction between the drug and the target leads to the activation of NF-κB (nuclear factor kappa-light-chain-enhancer of activated B cells), a transcription factor that induces the increased expression of a variety of cytokines, such as interferon α, TNF α (tumor necrosis factor α), interleukin (IL)-6, IL-8, IL-10, and G-CSF (granulocyte colony stimulating factor) [19].

In addition, 1% Tirb ointment is the most recently FDA- and EMA-approved ointment used to treat non-hyperkeratotic and non-hypertrophic AK of the face and scalp [18,20]. The active drug is Tirb at a 1% concentration. Tirb is an antiproliferative agent able to bind tubulin, likely in the same binding site as colchicine [21]. Once bound to its target, Tirb blocks tubulin polymerization during the formation of the mitotic spindle, leading to a cell cycle arrest in the G2/M phase. Such arrest causes the activation of apoptotic pathways such as PARP (poly(adenosine diphosphate [ADP]–ribose) polymerase) and caspase 3 cleavage. Moreover, Tirb induces p53 expression and inhibits Src kinase signaling [22]. Tirb binds tubulin in a totally reversible way, and this behavior is responsible for the significantly lower toxicity of the drug in comparison to the other tubulin inhibitors, i.e., podophyllotoxin, vinca alkaloids, colchicine, and its analogues [23].

### 2.2. Literature Search

#### 2.2.1. Efficacy Data

We searched the available scientific literature to standardize treatments and obtain efficacy data. We extrapolated complete clearance and partial clearance data from clinical studies, but in the pharmacoeconomic evaluation, we used only the values of complete clearance rates, defined as the proportion of patients at the endpoint with a count of zero lesions in the treatment area. In detail, we systematically searched peer-reviewed original articles published in English in the following databases: PubMed, Scopus, and Web of Science. No restriction in terms of time were set retrospectively. The first and the last searches were performed on September 2023 and March 2024, respectively. To perform the literature analysis, we combined the term “Actinic keratosis” with the terms: “5-fluorouracil”, “Diclofenac”, “Imiquimod”, or “Tirbanibulin” using the Boolean connector “and” (i.e., (Actinic keratosis) AND (Imiquimod)). Secondly, we assessed the abstracts of all potentially pertinent articles to check if they met the eligibility criteria. In particular, we included in our work studies published in English and in peer-reviewed journals, designed as randomized controlled trials, and reporting the complete clearance of AK as endpoint. For some treatments, more than one clinical study proved eligible, but in this case, the trial performed on the greatest number of patients was chosen. Two researchers (M.H. and L.F.) performed the search of efficacy data from the literature, and in case of discordance or doubt, they consulted a third researcher (D.A.).

#### 2.2.2. Costs

This analysis was conducted within the framework of the Italian NHS; it is assumed that the healthcare system bears the costs of treatments as a citizen would bear them; therefore, we used the prices displayed to the public.

Since AK is a basically chronic disease treated for long periods, the chosen timeframe is crucial to estimate the total cost per each treatment. For the duration of each treatment, the maximum recommended duration is assumed to allow a comparison among the different options; therefore, the cost of each single treatment was computed while accounting for its duration, providing the treatment-specific total cost. We assumed that all the patients under examination would carry out all the scheduled follow-up steps, i.e., including the first dermatological visit and the subsequent follow-up following the treatment-specific regimen scheme. Therefore, these expenses were not taken into consideration. The cost for the Italian NHS was calculated considering the prices reported by AIFA for each medicine.

The different treatment options were then examined in pairs; in the event that one therapy was more expensive but also more effective than the other or less expensive but less effective, an ICER analysis was also performed. The incremental cost-effectiveness analysis makes it possible to study the relationship between the variation in the cost and effectiveness of a drug with respect to the same parameters of the other.

$$ICER=\frac{C_{A}-C_{B}}{E_{A}-E_{B}}$$

The result of this evaluation is represented by the calculation of an incremental cost for an additional unit of health, meaning that the lower the result, the less expensive it is to obtain an increase in effectiveness.

## 3. Results

The results, in terms of costs, effectiveness data arising from the literature, and the calculated C/E ratios for the considered AK treatment regimens are summarized in Table 1.

### 3.1. Literature Search: Costs and Efficacy Data

The 0.5% 5-FU + 10% SA cut. sol. must be applied on AKs once a day until the lesions have been completely cleared or for up to a maximum of 12 weeks. During the treatment, some side effects can occur: Erythema, inflammation, burning, pain, and itch are considered common side effects; headache, excessive skin peeling, and ulcers can also occur, though significantly less often. The effectiveness of 0.5% 5-FU + 10% SA cut. sol. was reported in a multicenter, randomized, double-blind trial for comparison with its vehicle and DIC-HA in the treatment of AK. In this study, 470 patients with mild to moderate hyperkeratotic AK were randomly assigned to three groups: 187 patients received 0.5% 5-FU + 10% SA cut. sol. once daily, 185 patients received DIC- HA twice daily, and 98 patients received the placebo once daily. Treatments were administered until complete clearance of lesions occurred or for a maximum of 12 weeks. At week 20, complete clearance was 55%, 32%, and 15% of patients treated in the three groups, respectively [24]. One unit of 0.5% 5-FU + 10% SA cut. sol. costs EUR 60.69 and is enough to guarantee a complete therapeutic cycle. Considering such cost and the efficacy data reported above, a C/E ratio of EUR 1.10 was calculated (Table 1).

The 4% 5-FU cream must be applied on AK once a day for 4 weeks. Some side effects can be observed during the treatment: Irritation, pain, and inflammation on the application site and eye irritation are the most common. In a randomized, double-blinded, multicenter study involving 841 AK patients, 4% 5-FU cream was tested. In this trial, the cream was applied once a day for 4 weeks. At the end of the treatment, complete and partial clearance was achieved in 54% and in 80% of the treated patients, respectively [27]. The unitary cost of 4% 5-FU cream is EUR 72.99. According to the dosage scheme, one unit should be enough to complete a therapeutic cycle. Taking into account such cost and the efficacy data arising from the literature, a C/E ratio of EUR 1.35 was calculated (Table 1).

DIC 3% + HA 2.5% gel must be applied on the AK area twice a day for 60–90 days. Normally, 0.5 g of gel is enough for a single application on a 25 cm^2^ area. Side effects can occur: Skin rush, contact dermatitis, skin redness, inflammation, pain, and eczema are the most common [18]. A multicenter, randomized trial was performed on 502 patients to test the effectiveness in AK treatment of DIC 3% + HA 2.5% gel in comparison to a gel containing ingenol mebutate at a 0.015% concentration. A total of 247 patients received the application of DIC 3% + HA 2.5% gel twice daily for 17 weeks. The evaluated endpoints were complete and partial clearance of AK lesions. Although lower than that obtained for ingenol mebutate gel, complete and partial clearance were obtained in 24% and 43% of treated patients, respectively [25]. The unitary cost of DIC 3% + HA 2.5% gel is EUR 74.67, and one unit is enough to complete a therapeutic cycle. Considering such cost and the efficacy data reported above, a C/E ratio of EUR 3.11 was calculated (Table 1).

The 3.75% IMQ cream must be applied once a day before sleeping for two 2-week treatment cycles separated by a 2-week no-treatment interval [18]. Once the cream is applied, it is important to avoid contact with water for 8 h. After such time, the cream must be removed with water and mild soap. The most common side effects are inflammation and skin redness, dryness, and flaking and appearance of skin ulcers and viral infections [18]. The use of 3.75% IMQ cream in the treatment of AKs was proven effective. A multicenter, randomized, placebo-controlled clinical trial was performed on 479 patients affected by AK. They were treated according to the following scheme: 2 weeks of treatment, 2 weeks of rest, and 2 weeks of treatment. Overall, 36% and 59% of people treated with the cream containing IMQ 3.75% had a complete and a partial clearance of AK lesions, respectively [26]. One unit of 3.75% IMQ cream costs EUR 96.1 and is enough to complete a therapeutic cycle. Taking into consideration EUR 96.1 as the cost of a therapeutic cycle and 36% as complete clearance rate, a C/E ratio of EUR 2.67 was calculated (Table 1).

The 5% IMQ cream must be applied three times a week, before sleeping, for 4 weeks [18]. After each application, any contact with water must be avoided, and the cream must be removed after 8 h with water and mild soap. The effectiveness of 5% IMQ cream was reported. A multicenter phase III, randomized, double-blind, vehicle-controlled study was performed on 286 patients affected by AK. Patients were treated with the cream three times a week for 16 weeks, followed by an 8-week non-treatment period. A complete clearance of AK lesions was achieved in 57% of treated patients, while a partial clearance of AK lesions was obtained in 72% of treated people [28]. The most common side effects to occur during the treatment with 5% IMQ cream are pruritus, pain, redness, and burning in the application site as well as headache, asthenia, nausea, skin ulcers, increased susceptibility to infections, and muscle and joint pain [18]. The cost of one unit of 5% IMQ cream is EUR 76.56 and is enough for a complete therapeutic cycle. Considering such cost and the efficacy data reported above, a C/E ratio of EUR 1.34 was calculated (Table 1).

The 1% Tirb ointment must be applied once a day for one five-day therapeutic cycle. The effectiveness of Tirb 1% ointment was tested in a phase III clinical trials on a total of 351 patients affected by AK. Tirb 1% ointment was applied for five consecutive days, and the outcomes were evaluated at day 57. In the study, complete clearance was obtained in the 54% of patients, while partial clearance was reached in 76% of patients [29]. During the treatment with 1% Tirb ointment, some common side effects, i.e., erythema, crusting, ulcers, swelling, pain, pruritus, and vesicles formation, can appear. One unit of 1% Tirb ointment costs EUR 75.59 and is enough to complete a therapeutic cycle. Considering such cost and the efficacy data reported above, a C/E ratio of EUR 1.40 was calculated (Table 1).

### 3.2. Incremental Cost/Effectiveness Ratio (ICER) Analysis

To analyze three or more treatments, it is necessary to couple them into pairs and compare them pairwise; therefore, in Table 2 we classify the pairs of treatments according to the criteria of cost and effectiveness, dividing them into four possible categories: The first is less expensive and more effective than the second, and therefore, it is more convenient; the former is more expensive and less effective than the latter, so it should be discarded; the first is more expensive but also more effective than the second, and in this case, it is necessary to carry out an evaluation of the incremental cost/effectiveness ratio (ICER); the first treatment is less effective but also less expensive than the second, so also, in this case, it is necessary to carry out an evaluation of the ICER.

Having identified the couples for which the evaluation of the ICER was indicated, we proceeded to calculate it, as shown in Table 3, following the procedure described in the Methods Section.

## 4. Discussion

The prevalence of AK is very different among countries, with Australia accounting the highest rates worldwide (40–60% of White population older than 40 years) [30]. Among French and Austrian populations, the prevalence rates are 4.7% [31] and 31% [32], respectively. In Europe, the highest prevalence was reported by the Rotterdam Study (37%) [33]. In Italy, it is estimated that 27.4% of the Italian population over 30 is affected by AK [10]. These data highlight the enormous need for treatments for AK and the cost they represent for the NHSs and shows how important it is to carry out economic evaluations of topical treatments for AK. Our goal was to identify treatments with a better cost/effectiveness ratio and to estimate the cost of the gain in effectiveness in cases where the available information made it possible. Although the costs considered in this work refer to Italy, and in other countries, costs are likely significantly different, the results described herein could be usefully consulted by the National Health Systems of other countries.

We therefore estimated the total costs of the treatments, as shown in Table 1, by referring to the recommended maximum duration of each therapy. We chose this duration for standardization and to make possible the comparison between the different treatments. For each therapeutic option, we calculated the C/E ratio, i.e., the ratio between the reported cost and effectiveness. The more expensive the treatment, the higher the C/E ratio; the lower the effectiveness, the higher the C/E ratio. Conversely, the cheaper the treatment, the lower the C/E ratio; the higher the effectiveness, the lower the C/E ratio. This allowed us to classify the treatments in Table 2, which is divided into four columns. The first two are self-explanatory: In the first, the first treatment is less expensive and more effective than the second and therefore preferable. Furthermore, 4% 5-FU cream is less expensive and equally effective compared to 1% Tirb ointment. In the second column, the first treatment is less effective and more expensive than the second and therefore should be discarded. In detail, DIC 3% + HA 2.5% gel is less effective and more expensive than 4% 5-FU cream, while 3.75% IMQ cream is less effective and more expensive than 4% 5-FU cream, 1% Tirb. Ointment, and 5% IMQ cream. However, the third and fourth columns, which have treatments that are either less expensive but less effective or more expensive but more effective, deserve further study. The 5% IMQ cream is more expensive and more effective than 1% Tirb ointment. Moreover, DIC 3% + HA 2.5% gel is less expensive and less effective than both 3.75% and 5% IMQ creams and 1% Tirb ointment; the 4% 5-FU cream is less expensive and less effective than 5% IMQ cream. Finally, 0.5% 5-FU + 10% SA cut. sol. is less expensive and less effective than 5% IMQ cream.

The ICER estimate allowed us to evaluate the increase in cost effectiveness of each pair of treatments; however, from the first column of Table 2, it was clear that 0.5% 5-FU + 10% SA cut. sol. was immediately more convenient than all treatments, with the exception of 5% IMQ cream, which despite its slightly greater effectiveness has a significantly higher cost. The ICER shows that the modest gain in effectiveness has a cost of EUR 7.94 and is therefore too expensive (Table 3).

As mentioned above, we referred to short-term outcomes of the investigated therapies. Although we did not consider long-term outcomes for the evaluation of effectiveness, some interesting data arising from the literature are worthy of mention. In a clinical study for the treatment of actinic keratosis, a recurrence rate of 14.2% was obtained in patients treated with 0.5% 5-FU + 10% SA cut. sol. at 12 months after the end of the therapy [34]. In a randomized study, the recurrence of AK lesions was reported at 12 months after the end of the therapy in 43% and 14% for 5% 5-FU cream and 5% IMQ cream, respectively [35]. In another study, a recurrence rate of 57% was calculated for 1% Tirb ointment in a 12-month follow-up [36]. Recurrence is an important event that can lead to the necessity of repeated treatment and therefore to increased costs.

We suppose that this work could have a significant impact on clinical guidelines, at least in Italy.

Nevertheless, it is important to note that many other factors, including the difference in induction of side effects, could be essential in the choice of one treatment rather than another. In detail, the newly introduced 1% Tirb ointment was reported as responsible for fewer adverse effects compared to 0.5% 5-FU + 10% SA cut. sol., 4% 5-FU cream, DIC 3% + HA 2.5% gel, 3.75% IMQ cream, and 5% IMQ cream. Indeed, severe local reactions, including vesiculation or pustulation and erosion or ulceration, were less frequent with such treatment in comparison with other topical treatments for AK. Systemic adverse events were not common with 1% Tirb ointment [17]. It is worthy of note that the occurrence of side effects has a significant impact on the overall costs of a therapy, and for this reason, it could be interesting to perform further studies to evaluate also the cost/safety ratio. In the case of the considered therapeutic options, the side effects are mainly local, while systemic, dangerous side reactions are quite rare. Nevertheless, side effects can require further treatments, impact quality of life of the patients, and reduce their adherence to the therapy, finally generating a reduction in the effectiveness.

In other published international studies with the same objective of evaluating the most cost-effective treatment for AKs, the evaluation method was the same, i.e., the cost-effectiveness analysis followed by ICER. The perspective was that of the National Health System, and the efficacy data were collected from published literature. However, in one study (Colombo et al.), a one-way and multi-way sensitivity analysis was performed using a Monte Carlo simulation [37]. They (Colombo et al.) concluded that DIC 3% + HA 2.5% was less expensive (EUR 256) than MAL-PDT (EUR 320) and IMQ (EUR 342). Efficacy was similar and better for DIC 3% + HA 2.5% and MAL-PDT (0.813%) versus 0.734% for IMQ, respectively. Therefore, DIC 3% + HA 2.5% was the treatment of choice for AK and the surrounding tissue from a pharmacoeconomic point of view [37]. A cost-effectiveness analysis to compare 0.5% 5-FU + 10% SA cut. sol. with cryosurgery was performed in Spain with the perspective of the NHS. The authors identified in 0.5% 5-FU + 10% SA cut. sol. the best therapeutic option, as it was both more effective and cheaper than cryosurgery [38]. Another similar study was carried out in the United Kingdom, and the authors suggested 0.5% 5-FU + 10% SA cut. sol. as the best option [39]. Finally, a study performed in the Netherlands had the aim to compare the effectiveness and the cost of 5% 5-FU cream with 5% IMQ cream, ingenol mebutate (IM) 0.015%, and methyl aminolaevulinate photodynamic therapy (MAL-PDT) for the treatment of AK localized in the head and neck area. The results reported by the authors highlighted the 5% 5-FU cream as the dominant cost-effective choice [40].

A limitation of our study is that we did not conduct a sensitivity analysis of the data. For the comparison of study results, the entry into the market of new drugs does not allow a full comparison between the results of this work and others published previously.

As with many studies of this type, a limitation is represented by the data collection carried out using efficacy estimates from other studies, and therefore, it is important to take this limitation into account.

A further limitation of the study is not having a lambda parameter, i.e., a threshold beyond which the public decision maker is not willing to pay to obtain a gain in effectiveness. If this threshold were defined, the statement to accept or refuse certain treatments based on their ICER value would have more force.

In this work, we considered just the topical treatments for AK. Interestingly, further studies in the future could be performed to compare the above-discussed topical treatments with laser therapy, cryotherapy, curettage, surgery, and PDT.

There are several topical therapies for the treatment of AKs. The choice of the topical therapy could depend on many factors, such as the efficacy, the safety, the cost, the availability of the product, the experience of the doctor, and many others. Our considerations lead to the conclusion that the cost/effectiveness ratio of 0.5% 5-FU + 10% SA cut. sol. could be better than that of the other topical therapies included in our analysis, but although the economic factor is a really important factor for the treatment decision, many other features, both of the patients (e.g., age, comorbidities, and compliance) and of the disease (e.g., hyperkeratotic or normal AK, presence of a field cancerization rather than a single lesion, etc.), have to be considered by the dermatologists to choose the correct and most appropriate topical therapy for AK. Considering this, the treatment choice should be based on many factors, including the economic factors.

## 5. Conclusions

Many different pharmacological field-targeted treatments are available in Italy to treat AKs, i.e., 4% 5-FU cream, 0.5% 5-FU + 10% SA cut. sol., DIC 3% + HA 2.5% gel, 3.75% IMQ cream, 5% IMQ cream, and 1% Tirb ointment. For such therapeutic treatments, we herein reported the effectiveness data arising from the scientific literature and the total costs calculated by correcting the cost of a single treatment for its duration. Therefore, we performed a cost-effectiveness analysis by calculating the C/E ratio per each treatment, randomly divided them into pairs, and comparing them by calculating the ICER. This analysis allowed us to find 0.5% 5-FU + 10% SA cut. sol. as the best treatment, as it is more effective and less expensive than all the others, except for 5% IMQ cream. For the latter, we found that the modest gain in effectiveness has a cost of EUR 7.94 and is therefore too expensive, mainly if we consider the high prevalence of AK in Italy, affecting 27.4% of the over-30 population [20]. Although cost-effectiveness analysis should be crucial for the decision of the most appropriate therapeutic approach towards AK, many other features are worthy to be considered by the dermatologists, such as the occurrence of side effects. In this regard, it could also be interesting to perform a cost/safety evaluation of the available treatments.

## Figures and Tables

**Table 1:** Summary of treatment regimens, costs, effectiveness, and C/E ratios for each field-targeted treatment option.

Treatment	Time and Number of Applications	Dosage	Unitary Cost (EUR)	Total Costs (EUR)	Effectiveness		C/E Ratio ^3^ (EUR)
					**%CC ^1^**	**%PC ^2^**	
0.5% 5-FU ^4^ + 10% SA ^5^ cutaneous solution (25 mL)	1 application per day for up to 12 weeks	NR	60.69	60.69	55% [24]	NR ^6^	1.10
DIC ^7^ 3% + HA ^8^ 2.5% gel (90 g)	2 applications per day for 60–90 days	In an area of 25 cm^2^, it is recommended to apply 0.5 g of cream per administration.	74.67	74.67	24% [25]	43% [25]	3.11
3.75% IMQ ^9^ cream (28 sachets)	1 application per day for 2 weeks, then 2 weeks of suspension, and then again 1 application per day for 2 weeks.	The application of 1 sachet is indicated in an area of 20 cm^2^, up to a maximum of two sachets per day.	96.1	96.1	36% [26]	59% [26]	2.67
4% 5-FU cream (20 G)	1 application per day for 4 weeks.	NR	72.99	72.99	54% [27]	80% [27]	1.35
5% IMQ cream (12 sachets)	3 applications per week for 4 weeks.	NR	76.56	76.56	57% [28]	72% [28]	1.34
1% Tirb ^10^ ointment (5 sachets)	1 application per day for 5 consecutive days.	In an area of 25 cm^2^, the application of one sachet per day.	75.59	75.59	54% [29]	76% [29]	1.40

^1^ CC: complete clearance; ^2^ PC: partial clearance; ^3^ C/E: cost/effectiveness; ^4^ 5-FU: 5-fluorouracil; ^5^ SA: salicylic acid; ^6^ NR: not reported; ^7^ DIC: diclofenac; ^8^ HA: hyaluronic acid; ^9^ IMQ: imiquimod; ^10^ Tirb: tirbanibulin.

**Table 2:** Detail of the four treatment pairwise comparisons: less expensive (C_A_ − C_B_ < 0) and more effective (E_A_ − E_B_ >= 0); more expensive (C_A_ − C_B_ > 0) and less effective (E_A_ − E_B_ < 0); more expensive (C_A_ − C_B_ > 0) but more effective (E_A_ − E_B_ > 0); less expensive (C_A_ − C_B_ < 0) but less effective (E_A_ − E_B_ < 0).

C_A_ ^1^ − C_B_ ^2^ < 0 and E_A_ ^3^ − E_B_ ^4^ >= 0	C_A_ − C_B_ > 0 and E_A_ − E_B_ < 0	C_A_ − C_B_ > 0 and E_A_ − E_B_ > 0	C_A_ − C_B_ < 0 and E_A_ − E_B_ < 0
The first treatment is less expensive and more or equally effective and therefore preferable to the compared treatment.	The first treatment is more expensive and less effective and therefore to be refused compared to the compared treatment.	The first treatment is more expensive but also more effective; the absence of the dominance of one of the two alternatives highlights a trade-off and makes it necessary to evaluate the ICER 5.	The first treatment is less expensive but also less effective; the absence of the dominance of one of the two alternatives highlights a trade-off and makes it necessary to evaluate the ICER.
0.5% 5-FU 6 + 10% SA 7 cut. sol. 8—1% Tirb 9 ointment	DIC 10 3% + HA 11 2.5% gel—4% 5-FU cream	5% IMQ 12 cream—1% Tirb ointment	DIC 3% + HA 2.5% gel—3.75% IMQ cream
0.5% 5-FU + 10% SA cut. sol.—DIC 3% + HA 2.5% gel	3.75% IMQ cream—4% 5-FU cream		DIC 3% + HA 2.5% gel—5% IMQ cream
0.5% 5-FU + 10% SA cut. sol.—3.75% IMQ cream	3.75% IMQ cream—1% Tirb ointment		DIC 3% + HA 2.5% gel—1% Tirb ointment
0.5% 5-FU + 10% SA cut. sol. -4% 5-FU cream	3.75% IMQ cream—5% IMQ cream		4% 5-FU cream—5% IMQ cream
4% 5-FU cream—1% Tirb ointment			0.5% 5-FU + 10% SA cut. sol.—5% IMQ cream

^1^ C_A_: cost of treatment A; ^2^ C_B_: cost of treatment B; ^3^ E_A_: effectiveness of treatment A; ^4^ E_B_: effectiveness of treatment B; ^5^ ICER: incremental cost/effectiveness ratio; ^6^ 5-FU: 5-fluorouracil; ^7^ SA: salicylic acid; ^8^ Cut. sol.: cutaneous solution; ^9^ Tirb: tirbanibulin; ^10^ DIC: diclofenac; ^11^ HA: hyaluronic acid; ^12^ IMQ: imiquimod.

**Table 3:** The incremental cost-effectiveness analysis (ICER).

Treatment A	Treatment B	Incremental Cost (EUR)	Incremental Effectiveness	ICER ^1^ (EUR)
DIC ^2^ 3% + HA ^3^ 2.5% gel	1% Tirb ^4^ ointment	−0.92	−30	0.03
DIC 3% + HA 2.5% gel	5% IMQ cream	−1.89	−33	0.06
DIC 3% + HA 2.5% gel	3.75% IMQ ^5^ cream	−21.43	−12	1.79
0.5% 5-FU + 10% SA ^7^ cutaneous solution	5% IMQ cream	−15.87	−2	7.94
4% 5-FU ^6^ cream	5% IMQ cream	−156.69	−3	52.23

^1^ ICER: incremental cost/effectiveness ratio; ^2^ DIC: diclofenac; ^3^ HA: hyaluronic acid; ^4^ Tirb: tirbanibulin; ^5^ IMQ: imiquimod. ^6^ 5-FU: 5-fluorouracil; ^7^ SA: salicylic acid.

## Data Availability

Data are available within the article.
